# The association between family functioning and problem behaviors among Chinese preschool left-behind children: the chain mediating effect of emotion regulation and psychological resilience

**DOI:** 10.3389/fpsyg.2024.1343908

**Published:** 2024-02-27

**Authors:** Tianqi Qiao, Yan Sun, Pingzhi Ye, Jingfeng Yan, Xinxin Wang, Zhanmei Song

**Affiliations:** ^1^College of Education, Guangzhou University, Guangzhou, China; ^2^College of Teacher Education, Shaoxing University, Shaoxing, China; ^3^College of Education, Wenzhou University, Wenzhou, China; ^4^College of International Education, Wenzhou University, Wenzhou, China

**Keywords:** family functioning, problem behaviors, emotional regulation, mental resilience, preschool left-behind children

## Abstract

**Objective:**

The family environment has a significant impact on the psychological and behavioral development of children, especially those who are left behind in preschool and experience parent-child separation at a young age. These children face a greater risk of family dysfunction, which can lead to internalizing and externalizing problem behaviors. While numerous studies have established a connection between family functioning and problem behaviors, few have explored the underlying mechanisms driving this relationship. Our study seeks to address this gap by examining how emotion regulation and psychological resilience mediate the link between family functioning and problem behavior.

**Methods:**

The sample consisted of 940 preschool children (51.5% male, 48.5% female) with a mean age of 5.07 ± 0.80. The main guardians of the children were given the Family Assessment Device, Preschool Children’s Emotion Regulation Scale, the Devereux Early Childhood Assessment for Preschoolers (2nd edition), and the Social Skills Improvement System-Rating Scales to assess their family functioning, emotion regulation, psychological resilience, and problem behavior respectively.

**Results:**

Lower family functioning was associated with more severe problem behaviors in preschool left-behind children, and emotion regulation and psychological resilience partially mediated the relationship between family functioning and problem behaviors, respectively. In addition, emotion regulation and psychological resilience were also chain mediators between family functioning and problem behaviors.

**Conclusion:**

The study’s findings highlighted the crucial role of emotional regulation and psychological resilience in the correlation between family functioning and problem behaviors. It is recommended that policymakers and educators place a high priority on the cultivation of internal psychological resources, such as emotional regulation and resilience, in preschool-aged children when designing interventions to address problem behaviors.

## Introduction

The increase in urbanization in China has led to the migration of young and middle-aged rural laborers to cities. However, due to difficulties in transferring to other schools and high tuition fees, the children of rural laborers are forced to stay in the registered residence, becoming left-behind children (LBCs). LBCs refer to children aged 18 and under, whose parents have moved to other areas for more than half a year, who are left behind in their registered residence, and therefore cannot live with their parents (Cai et al., 2007). It is estimated that the number of LBCs in China was close to 69 million in 2015 (Lu et al., 2018). According to the *Report of *Rural Education Development in China (2020–2022)**, there were still 11.992 million LBCs in compulsory education in China in 2021 (Wu and Qin, 2022). Although the number of LBCs has decreased in recent years, they are still a large group (Lyu et al., 2022), and the education of these children will greatly influence China’s future.

The Chinese economy has developed rapidly in recent decades, but there still exists an unequal distribution of public resources, including education, healthcare, and employment, between urban and rural areas, especially for LBCs in rural areas (Jin et al., 2020). As a result of the prolonged absence of parental care, LBCs are less likely to obtain safe attachment, emotional support, self-regulation, and develop social skills (Jin et al., 2020). In the long run, they tend to exhibit a diverse range of externalizing problem behaviors, such as bullying, hyperactivity, aggressive behaviors (Zhang et al., 2019, 2021), and psychological problems, such as autism, unsociability, sensitivity, and suspicion (Luo et al., 2019). Problem behaviors can significantly impair children’s cognitive development, which is detrimental to physical and mental well-being and hinders healthy growth. LBCs’ problem behaviors have received widespread attention due to the specificity of the living environment and the large size of the group (Dong et al., 2019).

At present, the majority of studies on the problem behaviors of young children have been focused on school-age children, with few studies on preschool children (Dong et al., 2019; Jin et al., 2020; Wang et al., 2021). Research findings indicate that younger LBCs who experience prolonged separation from their parents are more susceptible to developing emotional and problem behaviors (Liu et al., 2009; Adhikari et al., 2014). If problems are not recognized or treated early, their emotional and behavioral disorders will worsen in adulthood, resulting in a number of negative outcomes, such as crime, drug abuse, mental health issues, and poor relationships (Fergusson et al., 2005). Therefore, greater emphasis should be placed on addressing the problem behaviors exhibited by preschool LBCs.

### Family functioning and problem behaviors

The most important change in the lives of LBCs compared to non-LBCs is the separation of parents and children. However, parent-child separation does not necessarily lead to children’s problematic behaviors. Parents (or one parent) working outside the home can improve socioeconomic status and bring new worldviews and outlooks on life, etc., and the associated changes in educational concepts may have a favorable impact on LBCs; however, they may also have negative effects, such as reduced parental behavioral control and monitoring, weakened parental support and guidance, which may have a negative impact on the LBCs (Wen and Lin, 2012). According to McMaster’s theory of family functioning model, the above positive and negative effects are actually related to the realization of family functioning in the process of family operation (Epstein et al., 1978). Family functioning (FF) refers to the effectiveness of family members in terms of family rules, family communication, emotional ties, and coping with external events (Olson et al., 1983), therefore, whether parents’ working outside the home has a negative impact on LBCs depends on whether it has caused damage to family functioning. Many researchers have examined the impact of FF on children’s problematic behaviors (PB) (van As and Janssens, 2002; Sikora et al., 2013; Stoutjesdijk et al., 2016). For example, Stoutjesdijk studied the correlation between FF and children’s classroom PB and revealed that poor FF was directly related to children’s classroom externalizing PB (Stoutjesdijk et al., 2016). Sikora’s study revealed a significant correlation between increased externalizing behaviors and diminished family functioning (Sikora et al., 2013). In addition, a literature review by van As and Janssens concluded that PB was strongly associated with low FF (van As and Janssens, 2002). Therefore, prior research has consistently indicated a strong association between FF and children’s PB (Sikora et al., 2013; Stoutjesdijk et al., 2016), while few researchers have explored the impact of FF on PB among LBCs.

The relationship between FF and PB can be conceptualized using the circumplex model and the McMaster model. The results-oriented circumplex model (Olson et al., 1983) shows that FF includes three dimensions: cohesion, adaptability, and communication. Scores on these three dimensions can be used as indicators of FF and can predict a child’s behavioral and psychological development. Previous studies have strongly demonstrated that children with more PB often come from families with poor cohesion, adaptability, and communication skills (Leidy et al., 2010; Coe et al., 2018). And, the process-oriented McMaster model (Epstein et al., 1978) indicates that the fundamental role of the family is to create a supportive environment that facilitates the holistic development of its members, encompassing physical, psychological, and social well-being. FF includes the structure of family relationships, flexibility of response, quality of association, and emotional connection of family members. A large number of studies have verified the McMaster model, showing that poor FF is associated with more serious PB (van As and Janssens, 2002; Sikora et al., 2013; Stoutjesdijk et al., 2016).

### Mediating effect of emotion regulation

Even though FF is linked to the PB of LBCs, external factors such as it cannot directly impact external behaviors but are mediated by internal factors of individuals. In previous studies on the influence factors of children’s PB, emotion regulation (ER) is considered to be an important intrapersonal factor (Eisenberg et al., 2010). ER to an individual’s capacity to flexibly respond to a range of positive or negative emotions in a way that is socially acceptable (Thompson, 1994). The emotional atmosphere in the family environment, the way of family expression, and the way of parenting have different degrees of influence on children’s ER (Morris et al., 2017). LBCs are confronted with a long-term separation from their parents, a lack of family education, and a lack of intellectual and emotional development, despite the fact that most of them are able to partially compensate for the effects of parental absence by being raised by other relatives. According to studies, the time and energy of family members caring for LBCs is mostly dedicated to work, with less focus on their education and development. Additionally, the social advancement of LBCs is still behind that of other children (Zhang et al., 2019, 2021). In a meta-analysis study, it was found that children’s emotional regulation was mainly dependent on their parents, who provided an environment that assisted them in self-regulating their inner feelings and behaviors (Li et al., 2019). The emotional climate and level of parent–child communication in LBCs were affected by the absence of parents and the limited energy of kinship caregivers, which led to poorer emotion regulation. Besides, ER is directly related to the PB of children. Research findings indicate that adaptive ER strategies exhibit a negative correlation with manifestations of depressive symptoms and anxious states, whereas maladaptive ER strategies show a positive correlation with manifestations of depressive symptoms and anxious states (Schäfer et al., 2017).

### Mediating effect of psychological resilience

Another important intraindividual factor that affects children’s PB is psychological resilience (PR) (Ding et al., 2023). PR is a personality, process, or outcome of an individual’s ability to move in a positive direction through continuous self-adjustment under adverse environmental stress (Campbell-Sills et al., 2006). Close family relationships and a good home environment are key protective factors for children to demonstrate good PR (Bonanno et al., 2015). After controlling for parent–child attachment and covariates at T1, a longitudinal follow-up study demonstrated that psychological resilience at T2 predicted high-quality children’s and adolescents’ parent–child attachment at T3. The psychological resilience of high-quality children is negatively impacted by low-quality parent-child attachment, leading to lower levels of future parent–child attachment (Zhang et al., 2023). The absence of parents from work can lead to changes in the family structure and weaken the emotional and educational functions of LBCs, which will inevitably impact their PR (Bonanno et al., 2015). PR serves as a safeguarding element for promoting emotional and behavioral health (Ding et al., 2023). According to studies, most preschool students have low PR, which prevents them from adapting to negative life events and results in a range of behavioral issues, including depression and anxiety (Wu et al., 2017; Ding et al., 2023). And low levels of PR predict more PB in the future (Wu et al., 2017).

### Chain mediating effect of emotion regulation and psychological resilience

In the existing literature, the majority of studies examined the individual effects of ER and PR on children’s PB, treating them as separate and independent variables (Eisenberg et al., 2010; Arslan, 2016). However, studies suggested that ER and PR had a positive correlation (Rodman et al., 2019; Zhang et al., 2021). Preschool is an important time for children and their emotional development in early childhood will profoundly affect their long-term psychological, emotional, and social adjustment (Darling-Churchill and Lippman, 2016). Children who have a better ability to regulate emotions are found to be more resilient to depression in childhood maltreatment (Rodman et al., 2019). Children with better ER often choose positive ER strategies, such as cognitive reconstruction, substitution, and self-consolation. Zhang found that children with more cognitive reconstruction strategies had more positive adaptive behaviors, children with more substitution behaviors had a higher level of PR, and children with more self-comforting behaviors had a higher level of PR (Zhang et al., 2021). Overall, LBCs with better ER have a lower risk of PR (Zhang et al., 2021).

### Purpose and research hypotheses

**Figure 1:**
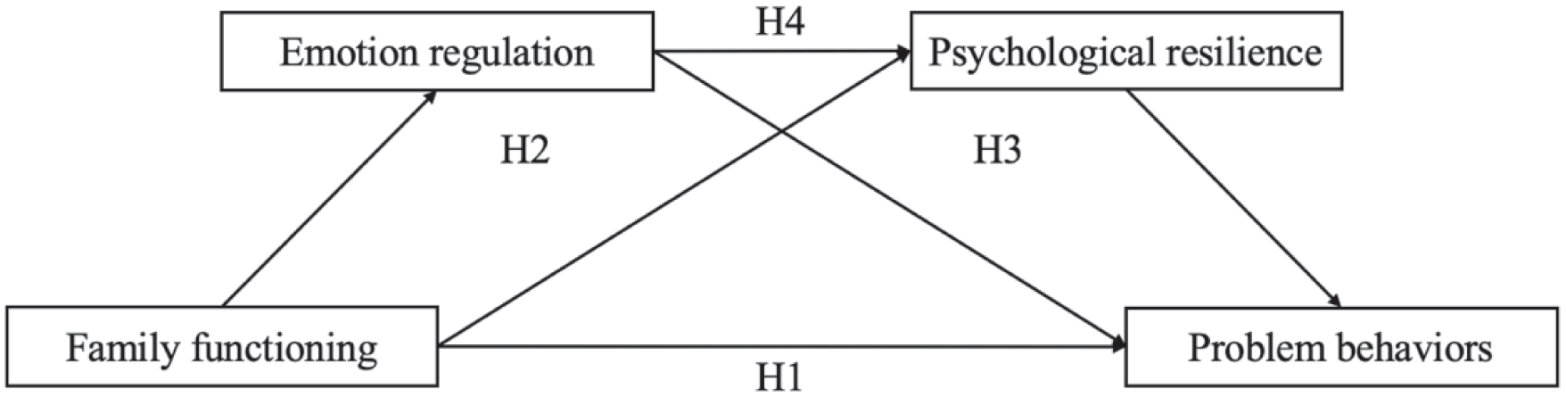
Hypothetical conceptual model.

While some studies have confirmed that ER and PR are associated with FF and PB in children, the specific influence of ER and PR on this relationship remains unclear. To the best of our knowledge, this study represents the initial exploration of the concurrent mediation effects of both ER and PR. Given the considerable population of preschool LBCs in China and the subsequent occurrence of PB resulting from early parental separation, finding effective measures to address these PB and prevent the development of more severe emotional and behavioral disorders in adulthood has become an urgent concern. The objective of the current research is to thoroughly explore the interactions among these four variables, thereby offering a theoretical foundation for enhancing the PB of preschool LBCs. On the basis of previous empirical studies, we established a chain mediation model (Figure 1). This study posits the following hypotheses:

*H1*: FF is significantly and negatively associated with PB for LBCs.

*H2*: ER mediates the relationship between FF and the PB of LBCs.

*H3*: PR mediates the relationship between FF and the PB of LBCs.

*H4*: ER and PR jointly mediate the relationship between FF and the PB of LBCs.

## Materials and methods

### Participants and procedure

This was a cross-sectional study that utilized the convenience sampling method to collect the data. In May 2023, questionnaires were distributed online to the primary guardians of left-behind children in nearly 30 kindergartens in the eastern province of China. In this study, the majority of the children’s primary guardians were the children’s uncles (30.6 percent of the total sample) and aunts (60.9% of the total sample), and a very small number of friends of the children’s parents (8.5% of the total sample). Informed consent was obtained from the primary guardians of all participating children, parents, and teachers, and the ethical principle of voluntary participation was communicated. According to the criteria for preschool left-behind children (Cai et al., 2007) (separated from parents for more than half a year; children who stayed to live in the household registration area; children’s age was 3–6 years old), questionnaires that did not meet the criteria for left-behind children were excluded, and 940 valid questionnaires were ultimately collected (recovery rate: 87.1%). The mean age of the study participants was 5.07 years (SD = 0.80, range = 4.00 ~ 6.00), including 484 boys (51.5%) and 456 girls (48.5%). The study protocol was reviewed through the review board of the host university to ensure compliance with the research ethics.

### Measures and instruments

#### Personal information form

The personal information form comprises the child’s age, gender, and whether he or she is a single child.

#### Family functioning

FF was measured using the subscale of the Family Assessment Device (FAD; Chinese Version) (Li et al., 2013). The scale was composed of 60 items and 7 factors (Epstein et al., 1983), and the factors included: problem-solving, communication, family role, emotional response, emotional involvement, behavioral management, and general function. The general functional factor consisted of 12 items that were strongly correlated with the other six factors, which could assess the health or morbidity of family functioning as a whole. The utilized scale adopts a 4-point Likert scale, spanning from 1 (strongly disagree) to 4 (strongly agree), where higher scores reflect enhanced FF. In the sample of Chinese preschool children, the study discovered that the general function scale had favorable reliability and validity (Bai et al., 2020; Almutairi et al., 2022). It has also been suggested that general functional factors alone can be used as a tool for assessing family functioning in general (Kabacoff et al., 1990; Ridenour et al., 1999). Therefore, this study used the general functional factor subscale, and the Cronbach’s *α* coefficient of the subscale was 0.845.

#### Emotion regulation

The Preschool Children’s Emotion Regulation Scale was used to measure the level of ER (Lu and Chen, 2007). The 36-item questionnaire was categorized into six domains: cognitive reconstruction, passive processing, problem-solving, alternative action, self-consolation, and venting. This scale was utilized to assess the employment of diverse emotional adjustment strategies when confronted with adverse situations. The total score is obtained by dividing the score of each dimension by the number of questions, as calculated after assessing each dimension (≤5 points). Better ER in children is indicated by a higher score on the positive emotion strategy. The scale has demonstrated good reliability and validity among Chinese preschool children (Zhang et al., 2021). The study yielded Cronbach’s *α* coefficients between 0.774 and 0.840 for the total scale and its respective subscales.

#### Psychological resilience

PR was measured using the Devereux Early Childhood Assessment for Preschoolers (DECA-P2; Second Edition) (LeBuffe and Naglieri, 2013), with 38 items. The scale has three dimensions: attachment/relationship, initiative, self-regulation, and behavioral problems. As a behavioral rating scale, the DECA-P2 was used to assess child protective factors that are critical to society, emotional health, and resilience, and to screen children for PB. Each item is rated on a scale from 0 (never) to 4 (always). A higher score indicates a higher level of PR in the child. The scale has demonstrated good reliability and validity among Chinese preschool children (Zhang et al., 2021). In this study, Cronbach’s *α* coefficients for both the overall scale and its subscales ranged from 0.88 to 0.96.

#### Problem behaviors

The measurement of PB was conducted using the subscale of Social Skills Improvement System -Rating Scales (SSIS-RS; Parental Version) (Gresham and Elliott, 2008). The scale consists of 33 items and 5 dimensions, including externalizing problems, bullying problems, hyperactivity problems, internalizing problems, and autism problems. Externalizing problem behaviors include verbal or physical aggression, an inability to control one’s temper, and arguments. Bullying problem behaviors include bullying others, forcing children to do things against their will, and intimidating them. Hyperactivity problems include being easily distracted or easily distracted. Internalizing problem behaviors include restlessness, frequent sadness, and low mood. Autistic problem behaviors include showing loneliness and avoiding others. A 4-point Likert scale is used by the questionnaire, with responses ranging from 1 (never) to 4 (always) based on the frequency of the child’s behaviors. The greater severity of problem behaviors in children is indicated by a higher score on the scale. Strong reliability and validity have been established for the scale when utilized in the Chinese population (Wang et al., 2021). In this study, the Cronbach’s *α* coefficients of the total scale and subscales ranged from 0.84 to 0.96.

#### Statistical analyses

Initially, descriptive statistics and Pearson correlation coefficients were calculated using the statistical software SPSS 24.0 to examine the key variables. Second, we employed MODEL 6 (Hayes, 2012) from the SPSS macro program PROCESS to construct a structural equation model. This model was utilized to investigate the underlying mechanism of influence between FF and PB. Next, the bias-corrected percentile bootstrap method, utilizing 5,000 samples and 95% confidence intervals (95% CIs), was employed to examine the significance of the indirect effect. Gender, age, and being an only child were incorporated as covariates in the model. Finally, the construction of competing models was done using Amos 26 statistical software for robust analyses.

## Results

### Common method bias test

The data for the four questionnaires on FF, ER, PR, and PB were collected from the same participants, which raises the possibility of common method bias. To reduce the potential bias, several measures were implemented, such as ensuring anonymity, including reverse items, emphasizing independent responses, and maintaining the confidentiality of information. However, it is important to acknowledge that despite these efforts, the potential covariation between the independent and dependent variables may have been influenced by the consistency of the testing environment and the homogeneity of participant sources. Therefore, after data retrieval, a test was conducted to examine the presence of common method bias.

To assess the potential presence of common method variance, Harman’s single-factor test was conducted by performing an unrotated exploratory factor analysis on all variables (Harman, 1970). The results revealed 17 factors with eigenvalues greater than 1. The variance explained by the first factor was 22.86%, which is less than the recommended threshold of 40% (Podsakoff et al., 2003). Thus, there was no significant common method bias in this study.

### Descriptive statistics and correlation analysis

Demographic variables such as the gender and age of the children as well as the main variables such as FF, ER, and PR were analyzed via Person correlation. As indicated in Table 1, the correlation coefficients between FF, ER, PR, and PB were found to be statistically significant. Based on the findings from the correlation analysis, FF exhibited a positive correlation with ER and PR (*p* < 0.01), but negatively correlated with PB (*p* < 0.01); A positive correlation was found between ER and PR (*p* < 0.01), and a negative correlation with PB (*p* < 0.01). Additionally, PR exhibited a negative correlation with PB (*p* < 0.01). In addition, child age was significantly associated with FF (*p* < 0.05), child gender was significantly associated with ER (*p* < 0.05) and PR (*p* < 0.01), and being an only child was associated with FF (*p* < 0.01) and PR (*p* < 0.05).

**Table 1:** Descriptive analysis and correlations.

	Mean	*SD*	1	2	3	4	5	6
1. Age	5.08	0.80	–					
2. Gender	1.48	0.50	0.04^**^	–				
3. Only child	1.76	0.43	0.13^**^	−0.06^*^	–			
4. Family functioning	37.53	5.59	−0.05^*^	0.02	−0.10^**^	–		
5. Emotion regulation	54.29	10.97	0.02	0.07^*^	0.01	0.42^**^	–	
6. Psychological resilience	98.54	21.10	0.01	0.10^**^	−0.07^*^	0.66^**^	0.65^**^	–
7. Problem behaviors	45.54	12.46	−0.02	−0.04	−0.04	−0.35^**^	−0.40^**^	−0.41^**^

*p < 0.05, **p < 0.01.

### Significance test of the mediating effect

**Figure 2:**
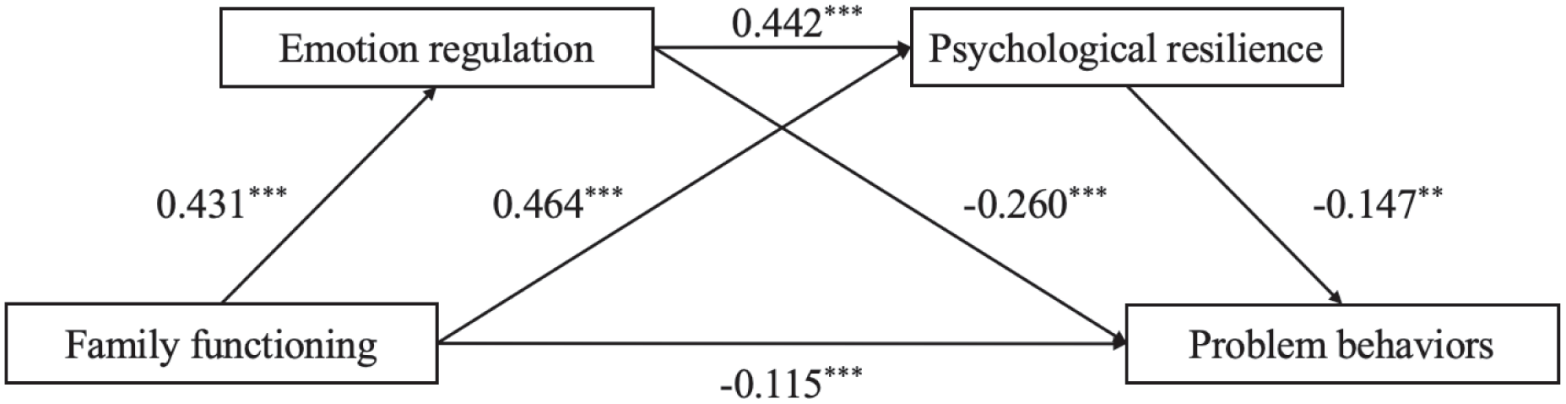
Chain mediation model.

After standardizing all the variables, Model 6 of the SPSS macro program PROCESS developed by Hayes was utilized to investigate the mediating effect of ER and PR in the relationship between FF and PB. The regression equation included age, sex, and only-child status as covariates. The results are presented in Table 2; Figure 2.

**Table 2:** Regression analysis of the relationship between family functioning and problem behaviors.

Regression equation		Fitting index			Significance	
Result variable	Predictor variable	*R*	*R*^2^	*F*	β	*t*
Problem behaviors		0.364	0.132	35.584^***^		
	Age				−0.013	−0.345
	Gender				−0.013	−0.204
	Only child				−0.199	−2.774
	Family functioning				−0.362	−11.789^***^
Emotion regulation		0.432	0.186	53.549^***^		
	Age				0.046	1.203
	Gender				0.117	1.948
	Only child				0.072	1.035
	Family functioning				0.431	14.413^***^
Psychological resilience		0.775	0.600	280.498^***^		
	Age				0.025	0.966
	Gender				0.112	2.678
	Only child				−0.075	−1.535
	Family functioning				0.464	20.153^***^
	Emotion regulation				0.442	19.432^***^
Problem behaviors		0.477	0.227	49.722^***^		
	Age				0.005	0.139
	Gender				0.042	0.713
	Only child				−0.186	−2.747^*^
	Family functioning				−0.155	−4.019^***^
	Emotion regulation				−0.260	−6.905^***^
	Psychological resilience				−0.147	−3.209^**^

All variables included in the model were standardized. The control variables consisted of age, gender, and being an only child. Family function served as the independent variable, while problem behaviors were the outcome variable. ^*^p < 0.05, ^**^p < 0.01, ^***^p < 0.001.

There was a significant negative correlation observed between FF and PB, and the direct path from FF to PB was found to be significant (*β* = −0.362, *p* < 0.001), supporting Hypothesis 1. FF was significantly and positively associated with ER (*β* = 0.431, *p* < 0.001) and PR (*β* = 0.464, *p* < 0.001), and ER was significantly and positively associated with PR (*β* = 0.442, *p* < 0.001). ER was significantly and negatively associated with PB (*β* = −0.260, *p* < 0.001), and there was a significant negative association between PR and PB (*β* = −0.147, *p* < 0.01). These results indicated that ER and PR have significant mediating effects on FF and PB, respectively. In addition, there was a significant chain mediation effect between ER and PR in FF and PB, and hypotheses 2–4 were supported.

Table 3 provides the unstandardized estimates for the overall effect, the direct effects of each indirect path, and the 95% confidence interval indicating the mediating effect. In Table 3, all three indirect paths have 95% confidence intervals that do not include zero, demonstrating significant chain mediating effects of ER and PR. The overall indirect effect size of FF on PB was determined to be −0.208. The mediating effect contributed to 57.30% of the total effect. The mediating effect comprised three indirect effects: FF → ER → PB (the mediating effect value is −0.112); FF → PR → PB (the mediating effect value is −0.068); FF → ER → PR → PB (the mediating effect value is −0.02). Hypotheses 2–4 were confirmed again.

**Table 3:** Emotion regulation and psychological resilience in the mediation effect analysis.

	Indirect effects	Boot SE	Boot LLCI	Boot ULCI	Relative mediation effect
Total indirect effect	−0.208	0.025	−0.258	−0.159	57.30%
Indirect effect 1	−0.112	0.024	−0.162	−0.067	30.85%
Indirect effect 2	−0.068	0.021	−0.108	−0.027	18.73%
Indirect effect 3	−0.028	0.009	−0.046	−0.011	7.72%

LL, lower limit; CI, confidence interval; UL, upper limit.

Indirect effect 1: family functioning → emotion regulation → problem behaviors.

Indirect effect 2: family functioning → psychological resilience → problem behaviors.

Indirect effect 3: family functioning → emotion regulation → psychological resilience → problem behaviors.

### Robust analysis

To determine whether the current model was the best fit for the data, we conducted robust analyses. Specifically, we compared the fit indices of the competing models (Model 1: Emotion Regulation → Psychological Resilience as the mediating variable; Model 2: Psychological Resilience → Emotion Regulation as the mediating variable). The results showed that Model 1 (fit indices: *χ*^2^/*df* = 2.816, CFI = 0.987, TLI = 0.980, AIC = 105.963, RMSEA = 0.056, SRMR = 0.018) fit better than Model 2(fit indices: *χ*^2^/*df* = 5.718, CFI = 0.970, TLI = 0.953, AIC = 169.793, RMSEA = 0.086, SRMR = 0.027). This finding suggested that the current model with emotion regulation → psychological resilience as the mediating variable was the best fit for the data.

## Discussion

Within this study, a chain mediation model was employed to provide additional confirmation of the direct association between FF and PB among preschool LBCs (van As and Janssens, 2002; Almutairi et al., 2022; Peng et al., 2023). Our findings revealed that both ER and PR played partial mediating roles in the association between FF and PB among preschool-aged LBCs. FF affected the PB of preschool LBCs through three pathways: ER, PR, and ER → PR, which helps us to further understand the relationship between FF and PB of preschool LBCs, and also helps kindergartens to properly adjust plan to further improve the externalizing and internalizing of PB of preschool LBCs.

### The relationship between FF and PB

This study revealed that lower FF was associated with more severe PB in preschool LBCs, which was consistent with the findings of previous studies (Sikora et al., 2013; Stoutjesdijk et al., 2016; Peng et al., 2023). For LBCs, parents out for work causes changes in the original family structure, leading to the weakening of the emotional and educational functions of families. Besides, it will inevitably have an important impact on the PB of LBCs.

### Independent mediating effect of ER

Consistent with the findings of prior research, this study discovered that ER serves as a mediating factor between FF and PB among preschool LBCs (Eisenberg et al., 2010; Thomsen and Lessing, 2020). ER acts as a bridge in this process. The influence of FF on preschool LBCs can be realized not only through direct influence on PB but also through indirect access to ER. This may be because the absence of parental roles leads to the impairment of FF, and the decline in the quality of the individual growth environment of preschool LBCs, which directly affects the formation of children’s ER (Chen et al., 2023). Low ER not only leads to the worsening of children’s original implicit or explicit problem behaviors, but also leads to depression and anxiety because they are unable to manage negative emotions (Ştefan and Avram, 2017; Thomsen and Lessing, 2020).

### Independent mediating effect of PR

Furthermore, this study revealed that PR serves as an intermediary factor between FF and PB among preschool-aged LBCs, aligning with previous research findings (Wu et al., 2017; Ding et al., 2023). PR also plays a role of bridge, and the influence of FF on the PB of preschool LBCs can be realized indirectly through PR. According to the family resilience theory (Walsh, 2003), families can provide social–emotional support for an individual’s PR. Close family relationships, good family communication, and the individual feeling accepted and loved are all very helpful for the development of positive self-concept and PR of family members. Based on the steeling effect theory (Rutter, 2012), individuals exhibiting higher levels of parental responsiveness (PR) may possess enhanced coping abilities in the face of stressful events and exhibit reduced sensitivity to negative experiences, ultimately leading to a decrease in problem behaviors.

### Chain mediating effect of ER and PR

Furthermore, the study results revealed that both ER and PR act as sequential mediators between FF and PB, which verifies the close relationship between ER and PR, that is, the ER of preschool LBCs also affected the development of PR, aligning with previous research findings (Rodman et al., 2019; Zhang et al., 2021). The higher the level of FF, the more family emotional support children receive, which is crucial for children to develop ER (Meyer et al., 2014). Research has shown that positive ER strategies, such as substitution, deflecting negative emotions, producing positive responses, and buffering some of the negative effects (Sattler and Font, 2018). However, the frequent use of poor ER strategies will make it difficult for children to recover from negative events, prevent them from overcoming the negative impact of negative events, and reduce the level of PR. Preschool LBCs themselves face premature separation from their parents. If individuals cannot overcome the negative effects of these situations, they are susceptible to experiencing internalizing problem behaviors, such as depression and anxiety, as well as externalizing problem behaviors, such as bullying and aggression (Ding et al., 2023).

## Limitations and implications

Several limitations exist within this study, which are outlined as follows: First, due to the limitations of relevant research methods, this study cannot infer causality. To address this issue in future research, longitudinal and experimental data may be employed to investigate the cause-and-effect relationship between variables, explore the dynamic relationship between variables, and further investigate the influence mechanism of FF on the PB of preschool LBCs. Second, although this study explored the mediating effect of individual factors (ER and PR), important variables closely related to PB, such as self-esteem and self-efficacy, should be further studied to fully explain the relationship model between FF and PB. Finally, considerable research has been conducted on the impact of grandparents serving as guardians for left-behind children (Zhou et al., 2021; Hu et al., 2023). However, little is known about how the family functioning of preschool-aged left-behind children is impacted by primary guardians such as uncles and aunts. It would be valuable to explore the potential effects of uncles and aunts as guardians of left-behind children in the future.

This study is of great significance. Firstly, this study constructed a chain mediation model to elucidate the connection between FF and PB of preschool LBCs, which is helpful for understanding how the family environment of preschool LBCs affects behavior development, and has important practical significance. Secondly, this study holds significant value in promoting interventions aimed at addressing PB among preschool LBCs. The findings of this study validate the negative predictive effect of FF on preschool LBCs, underscoring the critical role that FF plays in the behavioral development of preschool children. Interventions that focus on improving FF (problem-solving, communication, family roles, emotional responses, emotional engagement, and behavioral control) are therefore promising ways to reduce PB in children. Finally, the chain mediation model established in this study offers an alternative approach to mitigating PB among preschool LBCs, namely, by fostering the development of ER and PR in children. For example, carefully designed picture-book intervention courses and music interventions are adopted for LBCs, focusing on improving ER and PR, cultivating their own protective psychological resources, and helping preschool LBCs make better use of psychological resources to cope with the negative impact of adverse environment, which will effectively reduce the occurrence of PB.

## Conclusion

The purpose of this study was to examine the impact of family functioning on problem behaviors and its impact mechanism on Chinese preschool left-behind children. Lower family functioning was found to be linked to more severe problem behaviors in preschool left-behind children, and emotion regulation and psychological resilience partially mediated the link between family functioning and problem behaviors. In addition, this study revealed that emotion regulation and psychological resilience can play a chain mediating role between family functioning and problem behaviors. Policymakers and educators should address the development of intraindividual factors (emotion regulation and psychological resilience) while creating interventions for problem behaviors in Chinese LBCs in order to mitigate the negative consequences of poor family functioning in preschool LBCs.

## Data availability statement

The raw data supporting the conclusions of this article will be made available by the authors, without undue reservation.

## Ethics statement

The study involving human participants was approved by The Research Ethics Committee of Wenzhou University. Informed consent was obtained from the primary guardians of all participating children, their parents, and their teachers, and the ethical principle of voluntary participation was communicated.

## Author contributions

TQ: Conceptualization, Formal analysis, Methodology, Writing – original draft, Writing – review & editing. YS: Methodology, Validation, Writing – review & editing. PY: Writing – review & editing. JY: Writing – review & editing. XW: Funding acquisition, Writing – review & editing. ZS: Supervision, Writing – review & editing.

## References

[ref1] Adhikari R. Jampaklay A. Chamratrithirong A. Richter K. Pattaravanich U. Vapattanawong P. (2014). The impact of parental migration on the mental health of children left behind. J. Immigr. Minor. Health 16, 781–789. doi: 10.1007/s10903-013-9809-5 23546615

[ref2] Almutairi S. Scambler S. Bernabé E. (2022). Family functioning and dental caries among preschool children. J. Public Health Dent. 82, 406–414. doi: 10.1111/jphd.12475, PMID: 34545569

[ref3] Arslan G. (2016). Psychological maltreatment, emotional and behavioral problems in adolescents: the mediating role of resilience and self-esteem. Child Abuse Negl. 52, 200–209. doi: 10.1016/j.chiabu.2015.09.010, PMID: 26518981

[ref4] Bai R. Wang Z. Qi J. Long L. Liang J. Li H.. (2020). Effects of family function and temperament on behaviors of preschool children. Chinese J. Women Child Health Res. 31, 1040–1044. doi: 10.3969/j.issn.1673-5293.2020.08.011

[ref5] Bonanno G. A. Romero S. A. Klein S. I. (2015). The temporal elements of psychological resilience: an integrative framework for the study of individuals, families, and communities. Psychol. Inq. 26, 139–169. doi: 10.1080/1047840X.2015.992677

[ref6] Cai Y. Wang Q. Yang H. (2007). Investigation and correlative research of the left-behind young children in Huanggang area. Stud. Preschool Educ. 6, 3–7. doi: 10.3969/j.issn.1007-8169.2007.06.001

[ref7] Campbell-Sills L. Cohan S. L. Stein M. B. (2006). Relationship of resilience to personality, coping, and psychiatric symptoms in young adults. Behav. Res. Ther. 44, 585–599. doi: 10.1016/j.brat.2005.05.001 15998508

[ref8] Chen W. Zhang G. Tian X. Wang L. (2023). Psychometric properties and measurement invariance of the emotion regulation questionnaire in Chinese left-behind children. Curr. Psychol. 42, 8833–8843. doi: 10.1007/S12144-021-02155-Y

[ref9] Coe J. L. Davies P. T. Sturge-Apple M. L. (2018). Family cohesion and enmeshment moderate associations between maternal relationship instability and children’s externalizing problems. J. Fam. Psychol. 32, 289–298. doi: 10.1037/fam0000346, PMID: 29698005 PMC5926812

[ref10] Darling-Churchill K. E. Lippman L. (2016). Early childhood social and emotional development: advancing the field of measurement. J. Appl. Dev. Psychol. 45, 1–7. doi: 10.1016/j.appdev.2016.02.002

[ref11] Ding X. Liang M. Song Q. Su W. Li N. Liu H.. (2023). Development of psychological resilience and associations with emotional and behavioral health among preschool left-behind children. Soc. Psychiatry Psychiatr. Epidemiol. 58, 467–476. doi: 10.1007/s00127-022-02325-8, PMID: 35788881

[ref12] Dong B. Yu D. Ren Q. Zhao D. Li J. Sun Y.-H. (2019). The resilience status of Chinese left-behind children in rural areas: a meta-analysis. Psychol. Health Med. 24, 1–13. doi: 10.1080/13548506.2018.1487986, PMID: 29927309

[ref13] Eisenberg N. Spinrad T. L. Eggum N. D. (2010). Emotion-related self-regulation and its relation to Children's maladjustment. Annu. Rev. Clin. Psychol. 6, 495–525. doi: 10.1146/annurev.clinpsy.121208.131208, PMID: 20192797 PMC3018741

[ref9001] Epstein N. B. Bishop D. S. Levin S. (1978). The McMaster model of family functioning. J. Marital Fam. Ther. 4, 19–31. doi: 10.1111/j.1752-0606.1978.tb00537.x

[ref14] Epstein N. B. Baldwin L. M. Bishop D. S. (1983). The McMaster family assessment device. J. Marital. Fam. Ther. 9, 171–180. doi: 10.1111/j.1752-0606.1983.tb01497.x

[ref15] Fergusson D. M. Horwood L. J. Ridder E. M. (2005). Show me the child at seven: the consequences of conduct problems in childhood for psychosocial functioning in adulthood. J. Child Psychol. Psychiatry Allied Discip. 46, 837–849. doi: 10.1111/j.1469-7610.2004.00387.x, PMID: 16033632

[ref16] Gresham F. M. Elliott S. N. (2008). Social skills improvement system: Rating scales. Bloomington, MN: Pearson Assessments.

[ref17] Harman H. H. (1970). Modern factor analysis (University of Chicago Press, Chicago, IL).

[ref18] Hayes A. F. (2012). PROCESS: A versatile computational tool for observed variable mediation, moderation, and conditional process modeling [White paper]. Available at: http://www.afhayes.com/public/process2012.pdf

[ref19] Hu Q. Zhou Y. Dong P. Xu C. Zhang Q. (2023). Contributors to well-being of Chinese left-behind families: a dyadic perspective from family resilience and grandparent–grandchild relationship. Child Fam. Soc. Work 28, 646–658. doi: 10.1111/cfs.12991

[ref20] Jin X. Chen W. Sun I. Y. Liu L. (2020). Physical health, school performance and delinquency: a comparative study of left-behind and non-left-behind children in rural China. Child Abuse Negl. 109:104707. doi: 10.1016/j.chiabu.2020.104707, PMID: 32932062

[ref21] Kabacoff R. I. Miller I. W. Bishop D. S. Epstein N. B. Keitner G. I. (1990). A psychometric study of the McMaster family assessment device in psychiatric, medical, and nonclinical samples. J. Fam. Psychol. 3, 431–439. doi: 10.1037/h0080547

[ref22] LeBuffe P. A. Naglieri J. A. (2013). Devereux early childhood assessment for preschoolers second edition(DECA-P2): User's guide and technical manual Kaplan Early Learning Company.

[ref23] Leidy M. S. Guerra N. G. Toro R. I. (2010). Positive parenting, family cohesion, and child social competence among immigrant Latino families. J. Fam. Psychol. 24, 252–260. doi: 10.1037/a0019407, PMID: 20545398

[ref24] Li J.-B. Willems Y. E. Stok F. M. Deković M. Bartels M. Finkenauer C. (2019). Parenting and self-control across early to late adolescence: a three-level Meta-analysis. Perspect. Psychol. Sci. 14, 967–1005. doi: 10.1177/1745691619863046, PMID: 31491364

[ref25] Li R. Xu F. Ji L. Zhang W. (2013). Revision of family assessment device (FAD). China J. Health Psychol. (in Chinese). 21:5. doi: 10.13342/j.cnki.cjhp.2013.07.003

[ref26] Liu Z. Li X. Ge X. (2009). Left too early: the effects of age at separation from parents on Chinese rural children's symptoms of anxiety and depression. Am. J. Public Health 99, 2049–2054. doi: 10.2105/AJPH.2008.150474, PMID: 19762669 PMC2759782

[ref27] Lu L. Chen G. (2007). The development of preschool Children’s emotion regulation strategies. Psychol. Sci. 30:4. doi: 10.3969/j.issn.1671-6981.2007.05.048

[ref28] Lu L. Yan F. Duan C. Cheng M. (2018). Changing patterns and development challenges of child population in China. Popul. Res. 42, 65–78. doi: CNKI:SUN:RKYZ.0.2018-03-006

[ref29] Luo J. Zou J. Ji M. Yuan T. Sun M. Lin Q. (2019). Emotional and behavioral problems among 3-to 5-year-olds left-behind children in poor rural areas of Hunan province: a cross-sectional study. Int. J. Environ. Res. Public Health 16:4188. doi: 10.3390/ijerph16214188, PMID: 31671897 PMC6862179

[ref30] Lyu Y. Chow J. C.-C. Hwang J.-J. Li Z. Ren C. Xie J. (2022). Psychological well-being of left-behind children in China: text mining of the social media website Zhihu. Int. J. Environ. Res. Public Health 19:2127. doi: 10.3390/ijerph19042127, PMID: 35206315 PMC8871950

[ref31] Meyer S. Raikes H. A. Virmani E. A. Waters S. Thompson R. A. (2014). Parent emotion representations and the socialization of emotion regulation in the family. Int. J. Behav. Dev. 38, 164–173. doi: 10.1177/0165025413519014

[ref32] Morris A. S. Criss M. M. Silk J. S. Houltberg B. J. (2017). The impact of parenting on emotion regulation during childhood and adolescence. Child Dev. Perspect. 11, 233–238. doi: 10.1111/cdep.12238

[ref33] Olson D. H. Russell C. S. Sprenkle D. H. (1983). Circumplex model of marital and family systems: Vl. Theoretical update. Fam. Process 22, 69–83. doi: 10.1111/j.1545-5300.1983.00069.x, PMID: 6840263

[ref34] Peng S. Peng R. Lei H. Liu W. (2023). Family functioning and problematic behavior among secondary vocational school students: the mediating role of hope and the moderating role of perceived social support. Personal. Individ. Differ. 207:112156. doi: 10.1016/j.paid.2023.112156

[ref35] Podsakoff P. M. MacKenzie S. B. Lee J.-Y. Podsakoff N. P. (2003). Common method biases in behavioral research: a critical review of the literature and recommended remedies. J. Appl. Psychol. 88, 879–903. doi: 10.1037/0021-9010.88.5.879, PMID: 14516251

[ref36] Ridenour T. A. Daley J. Reich W. (1999). Factor analyses of the family assessment device. Fam. Process 38, 497–510. doi: 10.1111/j.1545-5300.1999.00497.x 10668625

[ref37] Rodman A. M. Jenness J. L. Weissman D. G. Pine D. S. McLaughlin K. A. (2019). Neurobiological markers of resilience to depression following childhood maltreatment: the role of neural circuits supporting the cognitive control of emotion. Biol. Psychiatry 86, 464–473. doi: 10.1016/j.biopsych.2019.04.033, PMID: 31292066 PMC6717020

[ref38] Rutter M. (2012). Resilience as a dynamic concept. Dev. Psychopathol. 24, 335–344. doi: 10.1017/S0954579412000028 22559117

[ref39] Sattler K. M. Font S. A. (2018). Resilience in young children involved with child protective services. Child Abuse Negl. 75, 104–114. doi: 10.1016/j.chiabu.2017.05.004, PMID: 28579076 PMC5711608

[ref40] Schäfer J. Ö. Naumann E. Holmes E. A. Tuschen-Caffier B. Samson A. C. (2017). Emotion regulation strategies in depressive and anxiety symptoms in youth: a meta-analytic review. J. Youth Adolesc. 46, 261–276. doi: 10.1007/s10964-016-0585-0, PMID: 27734198

[ref41] Sikora D. Moran E. Orlich F. Hall T. A. Kovacs E. A. Delahaye J.. (2013). The relationship between family functioning and behavior problems in children with autism spectrum disorders. Res. Autism Spectr. Disord. 7, 307–315. doi: 10.1016/j.rasd.2012.09.006

[ref42] Ştefan C. A. Avram J. (2017). Investigating direct and indirect effects of attachment on internalizing and externalizing problems through emotion regulation in a cross-sectional study. J. Child Fam. Stud. 26, 2311–2323. doi: 10.1007/s10826-017-0723-7

[ref43] Stoutjesdijk R. Scholte E. M. Swaab H. (2016). Impact of family functioning on classroom problem behavior of children with emotional and behavioral disorders in special education. J. Emot. Behav. Disord. 24, 199–210. doi: 10.1177/1063426615587262

[ref44] Thompson R. A. (1994). Emotion regulation: a theme in search of definition. Monogr. Soc. Res. Child Dev. 59:25. doi: 10.2307/1166137 7984164

[ref45] Thomsen T. Lessing N. (2020). Children's emotion regulation repertoire and problem behavior: a latent cross-lagged panel study. J. Appl. Dev. Psychol. 71:101198. doi: 10.1016/j.appdev.2020.101198

[ref46] van As N. M. Janssens J. M. (2002). Relationships between child behavior problems and family functioning: a literature review. Int. J. Child Fam. Welfare 5, 40–51. Available at: https://ugp.rug.nl/IJCFW/article/view/37568/35142

[ref47] Walsh F. (2003). Family resilience: a framework for clinical practice. Fam. Process 42, 1–18. doi: 10.1111/j.1545-5300.2003.00001.x 12698595

[ref48] Wang S. Hu B. Y. LoCasale-Crouch J. Li J. (2021). Supportive parenting and social and behavioral development: does classroom emotional support moderate? J. Appl. Dev. Psychol. 77:101331. doi: 10.1016/j.appdev.2021.101331

[ref49] Wen M. Lin D. (2012). Child development in rural China: children left behind by their migrant parents and children of nonmigrant families. Child Dev. 83, 120–136. doi: 10.1111/j.1467-8624.2011.01698.x 22181046

[ref50] Wu Z. Qin Y. (2022). Report of rural education development in China (2020–2022). Beijing: Science Press. 430.

[ref51] Wu Y.-L. Zhao X. Ding X.-X. Yang H.-Y. Qian Z.-Z. Feng F.. (2017). A prospective study of psychological resilience and depression among left-behind children in China. J. Health Psychol. 22, 627–636. doi: 10.1177/1359105315610811, PMID: 26490625

[ref52] Zhang H. Chi P. Long H. Ren X. (2019). Bullying victimization and depression among left-behind children in rural China: roles of self-compassion and hope. Child Abuse Negl. 96:104072. doi: 10.1016/j.chiabu.2019.104072, PMID: 31319239

[ref53] Zhang R. Wang L.-X. Datu J. A. D. Liang Y. Dou K. Nie Y.-G.. (2023). High qualities of relationships with parents and teachers contribute to the development of adolescent life satisfaction through resilience: a three-wave prospective longitudinal study. J. Happiness Stud. 24, 1339–1365. doi: 10.1007/s10902-023-00647-1

[ref54] Zhang J. Wu Y. Qu G. Wang L. Wu W. Tang X.. (2021). The relationship between psychological resilience and emotion regulation among preschool left-behind children in rural China. Psychol. Health Med. 26, 595–606. doi: 10.1080/13548506.2020.1849748, PMID: 33206569

[ref55] Zhou Y. Yu N. X. Dong P. Zhang Q. (2021). Dyadic associations between grandparent–child relationship quality and well-being in Chinese left-behind families: mediating role of resilience. J. Happiness Stud. 22, 1889–1904. doi: 10.1007/s10902-020-00300-1

